# Policies for Tobacco and E-Cigarette Use: A Survey of All Higher Education Institutions and NHS Trusts in England

**DOI:** 10.1093/ntr/ntz192

**Published:** 2019-10-05

**Authors:** Anna K M Blackwell, Daina Kosīte, Theresa M Marteau, Marcus R Munafò

**Affiliations:** 1 School of Psychological Science, University of Bristol, Bristol, UK; 2 Behaviour and Health Research Unit, University of Cambridge, Institute of Public Health, Cambridge, UK; 3 MRC Integrative Epidemiology Unit, University of Bristol, Bristol, UK; 4 National Institute for Health Research Bristol Biomedical Research Centre (NIHR Bristol BRC), Bristol, UK

## Abstract

**Introduction:**

There is an absence of evidence regarding the impact of treating tobacco smoking and vaping equivalently in workplace policies. We aimed to describe and compare smoking and vaping policies in acute nonspecialist NHS Trusts (*n* = 131) and Higher Education Institutions (HEIs) (*n* = 131) in England.

**Methods:**

We conducted a census of smoking and vaping policies through organizational websites searches and direct requests for information. We recorded whether and where smoking and vaping were permitted.

**Results:**

Smoking was prohibited indoors in all organizations. No NHS Trust permitted smoking freely outdoors, in contrast with 60% of HEIs. In 27% of NHS Trusts and 33% of HEIs smoking was permitted in designated areas, while in 73% of NHS Trusts and 8% of HEIs smoking was prohibited anywhere on site. Vaping was prohibited indoors in all NHS Trusts and all but one HEI, but permitted freely outdoors in 18% of NHS Trusts and 75% of HEIs. Vaping was permitted in designated outdoor spaces in 23% of NHS Trusts: 21% had areas shared with smokers; 2% had separate vaping areas. Vaping was permitted in designated outdoor areas in 18% of HEIs, all of which were shared with smokers. Vaping was prohibited anywhere on site in 54% of NHS Trusts and 6% of HEIs.

**Conclusions:**

Policies vary considerably in whether vaping and smoking are treated equivalently. Smoking policies in most HEIs should be reviewed to include more effective tobacco control approaches. Evidence is needed on the impact of imposing shared or separate spaces on vapers and smokers.

**Implications:**

This report provides a comprehensive review of smoking and vaping policies in two types of organization across England. It highlights key discrepancies between current public health recommendations for vaping and existing workplace policies, which often lead to smokers and vapers sharing spaces. The report identifies the need for evidence on the impact of imposing shared spaces on smokers and vapers to inform workplace policies that maximize public health benefit.

## Introduction

Smoking tobacco and other substances in England is regulated under the Health Act 2006, which prohibits smoking at all times in premises that are open to the public, or used as a place of work by more than one person, within enclosed or substantially enclosed areas. The National Institute for Health and Care Excellence^1^ public health guidelines (PH48) additionally recommend that acute, maternity and mental health NHS services develop policies for smoke-free grounds, which have been considered the “gold standard” to achieve for over a decade.^2^ These guidelines are reinforced by the Tobacco Control Plan for England.^3^ There are no equivalent additional recommendations concerning smoking in Higher Education Institutions (HEIs). Vaping is not included in the Health Act 2006.

Variation in smoking and vaping policies across organizations impact the air quality for people accessing different sites, as well as the success of smoking cessation and time spent taking breaks to smoke or vape. Public Health England (PHE) suggests that vaping and smoking should be considered as separate issues and that e-cigarettes should not be routinely included in smoke-free policies.^4^ This position has been echoed in a recent report by the Royal College of Physicians,^5^ who agree that allowing vaping on NHS Trust grounds can support smoke-free policy compliance by allowing vaping as an alternative to smoking. However, these positions are not prescriptive and some Trusts choose to include vaping in their local smoke-free policies.^6^ In these instances, smokers and vapers may use the same outdoor space or designated areas. 

In England, approximately 5% of people vape, which has remained stable since 2013,^7^ and the most common reason for doing so is smoking cessation.^8^ However, the relative advantages and disadvantages of vaping are debated by policy makers, health professionals, and researchers.^9^ Some believe vaping has positive benefits due to its potential to help people quit smoking.^3,10–12^ Others believe that there is not enough known about the long-term health impact of vaping to promote it, and public health concerns have been raised about the potential for vaping to encourage tobacco smoking, either by renormalizing smoking or by acting as a gateway to smoking among young people.^13,14^ A review of the evidence on vaping did not support these concerns, based on the continued decline in cigarette smoking, and confirmed a previous conclusion that vaping is less harmful than smoking and can play a useful role as a cessation aid.^15^

As many people choose to vape to assist them in stopping smoking, it is important to understand the impact of only permitting vapers to use spaces that have to be shared with smokers. In addition to the impact of exposure to second-hand smoke in these spaces, it is possible that exposure to smoking cues reduces vaping urges, which could undermine quit attempts or smoking abstinence in current and former smokers. However, it is also possible that exposure to vaping could encourage this behavior in smokers increasing the desire to reduce or quit smoking through vaping. This survey aimed to describe and compare smoking and vaping policies in NHS Trusts and HEIs in England to inform field studies designed to generate evidence on the impact of imposing shared or separate spaces on vapers and smokers within public sector organizations. 

## Methods

We conducted a census of acute nonspecialist NHS Trusts (*n* = 131)^16^ and HEIs in England, that is, universities funded by the Higher Education Funding Council for England (HEFCE) (*n* = 131),^17^ to collect information related to organizational smoking and vaping policies as part of descriptive survey. These two types of institution were selected as examples of large public sector organizations, with different levels of internal regulation regarding smoking and vaping, to explore variation both within and between types of organizations. Information was collected between August 2018 and January 2019. Information was extracted by the first and second authors (AKMB, DK) related to whether and where smoking and vaping were permitted, that is, inside buildings, outside buildings including details of exclusion zones directly outside entrances, windows, etc., and details of designated spaces available for smoking and/or vaping. Any ambiguity in responses was discussed between AKMB and DK to reach consensus. 

Information was obtained from organizational websites. When no information was identified online, or there was any ambiguity regarding how to code the information based on our criteria, we contacted organizations directly to request further details, either via members of organizational Health and Safety Teams (or other appropriate contacts identified online) or through formal Freedom of Information requests. One follow-up correspondence was sent if required. In cases where the information was not identified, policies were recorded as “unavailable.”

## Results

Policy information was obtained from all 131 acute nonspecialist NHS Trusts in England; however, 5% reported that they did not have information available on vaping. Policy information was obtained from 129 HEIs (98%): all had smoking policy information, 1% had no vaping information. Freedom of Information requests were sent to all NHS Trusts: 91% replied, information was retrieved from online policies for the remaining 9%. Information was retrieved from organizational websites for 55% of HEIs, the remaining were contacted directly to request information or additional clarification.

**Figure 1.**
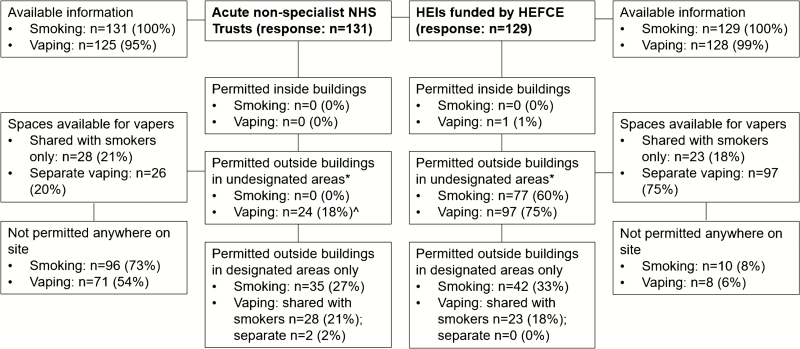
Flow chart of policies stipulating where smoking and vaping are permitted in acute nonspecialist NHS Trusts and Higher Education Institutions (HEIs) in England. HEFCE = Higher Education Funding Council for England. *Some restrictions apply (eg, directly outside entrances and windows or named areas); ^one Trust also has designated vaping areas.

Smoking was prohibited indoors in all NHS Trusts and HEIs in accordance with the Health Act 2006 (Figure 1), although some HEIs permitted smoking in designated student or residential accommodation. The ban on smoking indoors was extended to the areas directly outside buildings, for example, entrances and windows, across all NHS Trusts and most HEIs (96%), although 2% of HEI policies stated this behavior was discouraged rather than prohibited, and 1% allowed smoking outside Halls of Residence as an exception, as long as smoke did not drift back into buildings. Vaping indoors was prohibited across NHS Trusts. Vaping indoors was prohibited by almost all HEIs, although 1% allowed vaping indoors but only in offices or rooms not used by others, or on view to other staff or students. 

There was greater variation in policies related to smoking and vaping in outside spaces within organization grounds. Smoking was prohibited across all the NHS Trusts in outdoor areas, with the exception of designated spaces that could be used within the grounds at 27% of NHS Trusts. Some NHS Trusts noted that members of the public continued to smoke in smoke-free areas; while some aimed to redirect smokers to designated areas, others noted that policies were not enforced for the public, patients, or visitors. Vaping was prohibited in outdoor areas by the majority of NHS Trusts (77%), with the exception of designated vaping only areas (2%) or areas shared with smokers (21%) within the grounds (1% stated vapers could use the area adjacent to shelters). Vaping was allowed outside within the grounds in 18% of NHS Trusts, although 1% noted that vaping was not promoted. In 54% of NHS Trusts neither smoking nor vaping was allowed on site.

The majority of HEIs in England (60%) permitted smoking outdoors, although in 6% some restrictions applied and 1% allowed for separate local arrangements. In 33% of HEIs, smoking was only permitted in designated outdoor areas. Smokers were required to leave the site in 8% of HEIs. Vaping was permitted outdoors at 75% of HEIs: 12% within the grounds—although 2% included some exceptions—and an additional 63% when done away from entrances to buildings—although 4% included restrictions in certain areas and 1% allowed for separate local arrangements. Vaping was prohibited outdoors at 24% of HEIs, with the exception of designated spaces at 18% of HEIs that vapers could use with smokers. The scope of these designated areas varied from individual shelters to large areas, such as courtyards or terraces that would influence the extent to which vapers actually share the same space. In 6% of HEIs, neither smoking nor vaping was allowed on site.

Overall, smoking and vaping were treated as equivalent by the majority of NHS Trusts (76%) and HEIs (80%), while 20% of both NHS Trusts and HEIs treated smoking and vaping separately. However, in 4% of these HEIs the distinction was limited as smoking and vaping were both allowed outdoors (undesignated) but smokers must move away from buildings.

## Discussion

All acute nonspecialist NHS Trusts and HEIs surveyed in England complied with smoke-free legislation, which prohibits smoking indoors. Smoking was not permitted freely outdoors in any NHS Trusts. It was, however, permitted in designated spaces by 27%. This provision contravenes the 2013 NICE guideline (PH48), which recommends acute NHS Trusts have smoke-free grounds, reflecting the limited available evidence, which, although mixed, suggests that smoke-free outdoor policies could reduce smoking prevalence.^18^ Smoking was prohibited indoors across all HEIs and vaping indoors was prohibited by all but one HEI, where it was allowed in single use rooms. In the absence of guidelines, 60% permitted smoking outdoors within their grounds and 75% permitted vaping outdoors. The majority of NHS Trusts (76%) and HEIs (80%) treat smoking and vaping as equivalent, which is contrary to current recommendations to treat them separately and make vaping more accessible than smoking.^4,5,11^

Overall, there were separate spaces (designated or undesignated) that vapers could use away from smokers at 20% of NHS Trusts and 75% of HEIs. However, the extent to which smokers and vapers use separate spaces in practice remains unclear. For example, the nature of the local built environment may encourage smokers and vapers to share convenient spaces in close proximity to buildings, despite having the option to go elsewhere. In 24% of the HEIs and 80% of NHS Trusts, vapers had to use either designated spaces on site shared with smokers or to leave the grounds, which may lead to vapers and smokers sharing the nearest spaces available to main entrances. 

This survey builds on a recent House of Commons Science and Technology^11^ inquiry regarding e-cigarette use, including a survey of mental health NHS Trusts in England, which highlighted concerns about differences between NHS Trusts, leading the Committee to recommend a central policy allowing vaping by default. The Government agreed that NHS England should provide guidance permitting existing vapers to use e-cigarettes, and the option for existing smokers to use e-cigarettes as part of smoking cessation programmes.^19^ Another recent study of smoke-free policies in mental health NHS Trusts in England identified increasing support for vaping, including within private rooms, but also noted discrepancies between Trusts in the spaces available.^20^ Many organizations included in our current survey indicated that policies were under review, or due for review soon; specifically, three NHS Trusts stated that they anticipated allowing vaping on site, or allocating dedicated vaping points on site, in the future. Furthermore, by 2019/2020, the entire NHS estate will become smoke-free in line with the NHS England 5-year plan,^21^ and this provides the necessary scope for organizations to consider making vaping more accessible than smoking.

The degree of variability regarding where smoking and vaping are permitted within grounds of different organizations are not only issues for NHS Trusts and HEIs. Other organizations such as local authorities^22^ have extended the ban on smoking in enclosed spaces to include all spaces within their grounds and prohibit employees smoking within their working time. Public and private sector organizations should review their internal policies in line the latest evidence on the potential of e-cigarettes to reduce smoking.^12^ While policies should consider the potential nuisance of vaping to others, vaping should not be routinely included in smoke-free requirements.^4^ Separate spaces for vapers would protect them from second-hand tobacco smoke. Making these spaces more accessible than those for smoking could also support compliance with smoke-free policies and help promote smoking cessation.^4,5^

## Conclusions

In keeping with legislation in England, none of the institutions included in this survey permitted smoking cigarettes indoors. A minority of Trusts permitted smoking in designated spaces outside with most prohibiting smoking anywhere on site. By contrast, the majority of HEIs permitted smoking anywhere outdoors. Smoking policies in most HEIs should be re-drafted to include more effective approaches to tobacco control, as reflected in policies of acute NHS Trusts.

In the absence of specific legislation, policies for vaping are less consistent. The majority of NHS Trusts (76%) and HEIs (80%) treat vaping as equivalent to smoking, permitting or prohibiting both in the same manner and in the same spaces, although many HEIs allow both outdoors so these spaces are not necessarily confined. In contrast, 20% of NHS Trusts and HEIs consider smoking and vaping separately. Policy variation in whether, and to what extent, vaping and smoking are treated equivalently is linked, at least in part, to an absence of evidence on the consequences of this. Evidence is needed on the impact of imposing shared or separate spaces on vapers and smokers, to inform both local and national policies.

## Funding

This work was supported by a Collaborative Award in Science from the Wellcome Trust (Behaviour Change by Design: 206853/Z/17/Z) awarded to Theresa M. Marteau, Paul Fletcher, Gareth Hollands, and Marcus R. Munafò. The funder is not involved in the study design, data collection and analysis, decision to publish, or preparation of the manuscript.

## Declaration of Interests

None declared.
